# ﻿*Opacoptera* Gozmány (Lepidoptera, Lecithoceridae) from China, with descriptions of four new species

**DOI:** 10.3897/zookeys.1158.100396

**Published:** 2023-04-21

**Authors:** Shuai Yu, Shuxia Wang

**Affiliations:** 1 School of Life Sciences, Liaocheng University, Liaocheng 252000, China Liaocheng University Liaocheng China; 2 College of Life Sciences, Nankai University, Tianjin 300071, China Nankai University Tianjin China

**Keywords:** Gelechioidea, identification key, Lecithocerinae, new record, taxonomy

## Abstract

The genus *Opacoptera* Gozmány, 1978 is reviewed. Four species are described as new: *O.condensata***sp. nov.**, *O.hybocentra***sp. nov.**, *O.introflexa***sp. nov.** and *O.longissima***sp. nov.***Opacopterakerastiodes* Park, 2021 is newly recorded for China. Images of adults are provided, along with a key to the males of all the known species.

## ﻿Introduction

*Opacoptera* is a small genus classified in the subfamily Lecithocerinae. Gozmány (1978) established the genus for the single species *Lecithoceracallirrhabda* Meyrick, 1936 from China, the type species. Species of *Opacoptera* are known only from the Oriental Region. Wu and Liu (1992) and Wu (1996) described two Chinese species in the genus, *O.flavicana* Wu & Liu, 1992 and *O.ecblasta* Wu, 1996. Park and Kim (2021) described an additional species, *O.kerastiodes* Park, 2021, from Thailand. *Opacoptera* currently comprises four described species.

The aim of this paper is to review the genus *Opacoptera* and to describe four new species.

## ﻿Materials and methods

Specimens were collected in China since 1998 using light traps. Wingspan was measured from the tip of the left forewing to the tip of the right forewing. Genitalia slides were prepared following the methods introduced by Li (2002). All images were captured with digital microscopes (Leica M205A and Leica DM750), coupled with the Leica Application Suite 4.2 software. Terminology follows Gozmány (1978). The male and female genitalia are described from the ventral view.

All the specimens examined, including the type series of the new species, are deposited in the Insect Collection of Nankai University, Tianjin, China (NKU).

### ﻿Abbreviations

**NHMUK**Natural History Museum, London, United Kingdom

**IZCAS**Institute of Zoology, Chinese Academy of Sciences, Beijing, China

**NKU**Insect Collection of Nankai University, Tianjin, China

**TD** Type depository

**TL** Type locality

**ZMUC**Zoological Museum, Natural History Museum of Denmark, Copenhagen, Denmark

## ﻿Taxonomic accounts

### ﻿ Opacoptera

Taxon classificationAnimaliaLepidopteraLecithoceridae

Gozmány, 1978

2E501308-7984-58C8-B6D2-6BC207EA17FF

Opacoptera Gozmány, 1978. Type species: Lecithoceracallirrhabda Meyrick, 1936, by monotypy.

#### Generic characters.

**Figures 1, 2.**
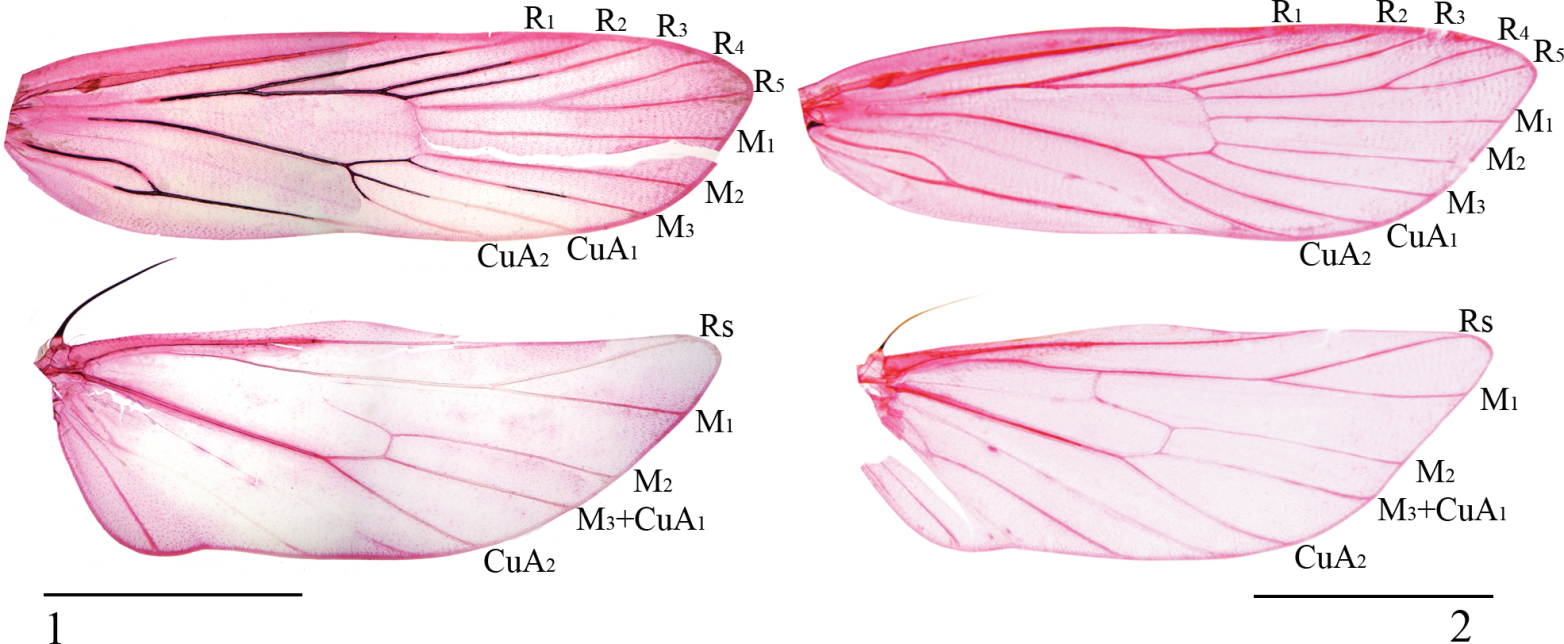
Wing venation of *Opacoptera* spp. **1***O.condensata* sp. nov., holotype, male, slide No. LSR12090 **2***O.hybocentra* sp. nov., paratype, male, slide No. YS19431. Scale bars: 2.0 mm.

Antenna as long as or slightly longer than forewing. Labial palpus with second palpomere thickened, third palpomere slender. Forewing narrowly elongate, usually dark brown with black patches; R_1_, R_2_ and R_3_ free, R_4_ and R_5_ stalked, M_1_, M_2_ and M_3_ separate, CuA_1_ and CuA_2_ short-stalked (Fig. 1) or separate (Fig. 2). Hindwing trapezoidal; M_2_ present, M_3_ and CuA_1_ coincident. All abdominal tergites with zones of spiniform setae.

Male genitalia. Cucullus narrowed, usually with a ventrobasal process. Juxta with horn-shaped or needle-like posterior lobes extending dorsad and smaller posterolateral lobes extending laterally. Aedeagus with two dorsal denticles; cornuti spiniform or needle-like.

Female genitalia. Antrum sclerotized along posterior margin forming a band. Signa of corpus bursae usually consisting of several teeth and a denticulate plate.

#### Diagnosis.

The genus is close to the monotypic genus, *Monerista* Meyrick, 1925. It can be distinguished by the trapezoidal hindwing without streak; whereas in *Monerista*, the lanceolate hindwing has a thinly scaled submedian streak (Clarke 1965: 183, fig. 1)

#### Distribution.

China (Gozmány 1978; Wu and Liu 1992; Wu 1996), Thailand (Park and Kim 2021).

#### Remarks.

The generic characters of *Opacoptera* were originally defined by Gozmány (1978) based on the type species, *Opacopteracallirrhabda* (Meyrick, 1936). Wu (1996) gave the female characters of the genus. In this paper, we revised the generic characters including the forewing venation, male and female genitalia after examining all species of the genus.

### ﻿Key to the males of *Opacoptera*

**Table:** 

1	Forewing pale, yellowish brown; juxta without posterior lobes (Fig. 6)	***O.flavicana***
–	Forewing dark; juxta with posterior lobes	**2**
2	Apices of posterior lobes of juxta extending posteriorly well beyond middle of tegumen (Fig. 17)	***O.longissima* sp. nov.**
–	Apices of posterior lobes of juxta extending posteriorly to anterior margin of tegumen or only slightly beyond	**3**
3	Inner margin of base of posterior lobes of juxta with medial, triangular projection (Fig. 15)	***O.introflexa* sp. nov.**
–	Posterior lobes of juxta smooth on inner margin	**4**
4	Posterior lobes of juxta needle-like; uncus longer than width (Fig. 13)	***O.ecblasta***
–	Posterior lobes of juxta horn-shaped; uncus wider than length	**5**
5	Length of ventrobasal process on cucullus greater than basal width of cucullus (Fig. 14)	***O.hybocentra* sp. nov.**
–	Length of ventrobasal process on cucullus less than basal width of cucullus	**6**
6	Cucullus widened or dilated apically (Fig. 16)	***O.kerastiodes***
–	Cucullus slightly narrowed apically	**7**
7	Juxta incised in triangle at middle on posterior margin; posterior lobes straight (Fig. 11)	***O.callirrhabda***
–	Juxta broadly concave on posterior margin; posterior lobes curved laterally at middle (Fig. 12)	***O.condensata* sp. nov.**

### ﻿ Opacoptera callirrhabda

Taxon classificationAnimaliaLepidopteraLecithoceridae

(Meyrick, 1936)

470B641C-A04A-5271-9E65-C3C530657B5E

Figs 3 , 11 , 18

Lecithocera callirrhabda Meyrick, 1936: 158. TL: China (Yunnan). TD: NHMUK.
Opacoptera callirrhabda (Meyrick): Gozmány, 1978: 179.

#### Material examined.

**China**: 1♀, 30.vii.2014, 3♂, 2–3.viii.2014, Yunnan, Dali, Mt. Weibao, 2205 m, KJ Teng et al. leg., slide nos. YS19428♂, YS19435♀.

#### Diagnosis.

This species is diagnostic in the male genitalia by the juxta incised at middle in a triangle on the posterior margin (Fig. 11). It is similar to *O.condensata* sp. nov., and the differences between them are stated in the diagnosis of the latter species.

#### Description.

**Figures 3–10.**
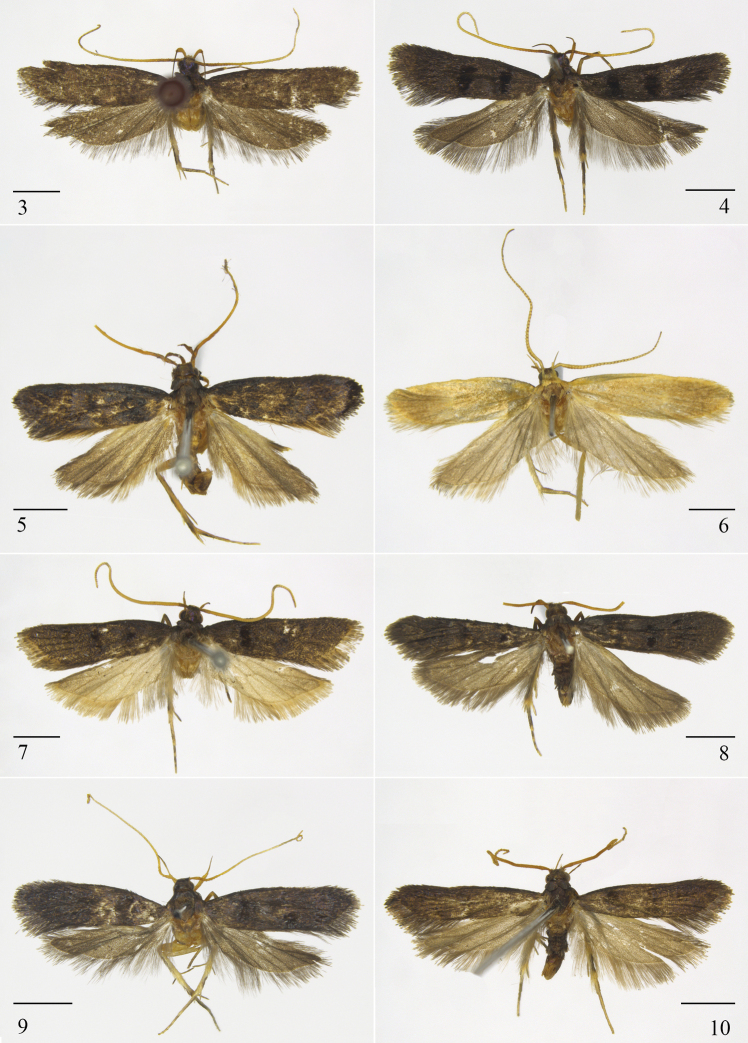
Dorsal habitus of *Opacoptera* spp. **3***O.callirrhabda* (Meyrick, 1936), female, YS19435 **4***O.condensata* sp. nov., paratype, female, LSR13198 **5***O.ecblasta* Wu, 1996, male, LSR11240 **6***O.flavicana* Wu & Liu, 1992, female, LSR11247 **7***O.hybocentra* sp. nov., holotype, male, YS19424 **8***O.introflexa* sp. nov., paratype, male **9***O.kerastiodes* Park, 2021, male, YS19048 **10***O.longissima* sp. nov., paratype, male. Scale bars: 2.0 mm.

Wingspan 13.5–15.0 mm (Fig. 3).

***Female genitalia*** (Fig. 18). Eighth abdominal sternite obtuse on posterior margin. Apophyses posteriores twice length of apophyses anteriores. Antrum cup-shaped, wrinkled, membranous except sclerotized along posterior margin forming a band. Ductus bursae subelliptical, slightly wider than corpus bursae, partly wrinkled anteriorly; ductus seminalis broad basally, slender distally. Corpus bursae elliptical; signa placed medially, consisting of five teeth in a longitudinal row and a small rounded, denticulate plate.

#### Distribution.

China (Sichuan, Yunnan) (Gozmány 1978; Wu 1996).

#### Remarks.

Meyrick (1936) described this species based on five male specimens (one from Shandong, two from Shaanxi and Yunnan, respectively) and placed it in the genus *Lecithocera*. Clarke (1965) assigned one of the specimens from Yunnan as the lectotype according to the original description. Gozmány (1978) noted that only the two specimens from Yunnan were conspecific. He also hypothesized that they were distinct from *Lecithocera*, so diagnosed the genus *Opacoptera* and placed the species therein. Wu (1996) was first to described the female of *O.callirrhabda* based on specimens with associated males collected at different times and from different localities of Sichuan. According to the description and the drawing given by Wu (1996), the female genitalia of *O.callirrhabda* has a narrowed ductus bursae and a single signum. However, the female of the species examined in this study from Yunnan is quite different, and we describe it herein.

### ﻿ Opacoptera condensata

Taxon classificationAnimaliaLepidopteraLecithoceridae

Yu & Wang sp. nov.

5521E36C-BDC8-59F8-86FD-4886148E320D

https://zoobank.org/BC7D6FE9-EABC-4BE8-B186-0A95C9CA359B

Figs 4 , 12 , 19

#### Type material.

***Holotype*: China**: ♂, Chongqing, Mt. Simian (29°19'N, 106°22'E), 1280 m, 14.vii.2012, YH Sun & AH Yin leg., slide No. LSR12090♂. ***Paratypes*: China**: 9♂1♀, 1280 m, 12–14.vii.2012, 9♂2♀, 900 m, 18–19.vii.2012, same locality and collector as holotype, slide No. YS19556♂; 12♂3♀, Zhejiang, Mt. Tianmu, 555 m, 3–6.vii.2014, AH Yin, XM Hu & QY Wang leg., slide No. YS19427♂; 1♂, Jiangxi, Xiaoxidong, 8.vii.1978, slide No. ZYM06008; 25♂, Henan, Neixiang, Xiaguan, 650 m, 10–12.vii.1998, HH Li et al. leg., slide Nos. LSR11295, ZYM05011, ZYM05052, ZYM0505, ZYM05054, ZYM06202, ZMR09020; 2♂2♀, Hubei, Zhuxi County, Quanxi Town, 868 m, 10–11.vii.2017, WD Qi et al. leg., slide Nos. YS17039♀, YS17040♀, YS17041♂, YS17042♂; 3♀, Hubei, Zhuxi County, Mt. Bagua, 790 m, 12–13.vii. 2017, WD Qi et al. leg., slide Nos. YS17055, YS17056, YS17057; 4♂4♀, Yunnan, Weishan County, Mt. Weibao, 2200 m, 20.vii.2001, HH Li & XP Wang leg., slide Nos. ZMR09017♂, ZYM06092♂, ZYM06198♂, ZYM06199♀; 5♂2♀, Yunnan, Weishan County, Mt. Weibao, 2244 m, 22–24.vii.2013, SR Liu, YQ Wang & KJ Teng leg., slide Nos. LSR13197♂, LSR13198♀; 12♂4♀, Shaanxi, Houzhenzi, 1330 m, 23–24.vii.2018, YY Li & JL Zhuang leg., slide No. YS19433♂, YS19434♀.

#### Diagnosis.

The male genitalia of the new species is similar to that of the type species, *O.callirrhabda* (Meyrick, 1936). It can be distinguished by the forewing having two black patches, CuA_1_ stalked with CuA_2_, and ductus seminalis having dense granules; in *O.callirrhabda*, the forewing has no black patches, CuA_1_ and CuA_2_ are separate, and the ductus seminalis has no granules.

#### Description.

Wingspan 13.0–15.0 mm (Fig. 4). Head dark brown. Antenna yellowish brown in basal 1/4, pale yellow in distal 3/4. Labial palpus yellowish brown, paler on inner surface; third palpomere as long as second palpomere. Thorax and tegula dark brown. Forewing dark brown, with two large, elliptical, black patches anteriorly reaching anterior margin of discal cell: first at basal 1/4, posteriorly reaching fold; second at middle, posteriorly reaching above dorsum; fringe dark brown; CuA_1_ and CuA_2_ short-stalked. Hindwing and fringe greyish brown; fringe with an orange white basal line.

***Male genitalia*** (Fig. 12). Uncus subcrescent. Gnathos with basal plate bell-shaped, bearing a papillary process at middle on posterior margin; median process wide in basal 1/3, narrowed to distal 2/5, thereafter slender to pointed apex, curved ventrad at distal 1/4 by a right angle. Valva wide in basal 1/4, narrowed slightly to cucullus; cucullus about half length of valva, narrowed slightly to obtusely oblique apex, costal margin shallowly concave except convex at base, ventrobasal process subtriangular, with a rounded apex; costal bar narrow, slightly expanded dorsad medially; sacculus a broad band, about 1/4 length of ventral margin of valva. Saccus rounded on anterior margin. Juxta broadly concave on posterior margin, denticulate along lateral sides of concavity; with a trapezoidal process at middle on anterior margin; posterior lobe strongly horn-shaped, curved outward at middle; posterolateral lobe short, narrow, extending laterally. Aedeagus nearly as long as valva, slightly widened medially, dorsal denticles larger than cornuti; cornuti consisting of 1–3 conic spines.

***Female genitalia*** (Fig. 19). Eighth abdominal sternite convex on posterior margin. Apophyses posteriores twice the length of apophyses anteriores. Antrum subrectangular, wrinkled, membranous except sclerotized along posterior margin forming a band. Ductus bursae narrowed posteriorly, widened distinctly toward corpus bursae, with diffused granules near base of ductus seminalis; ductus seminalis broad, with dense granules on inner wall. Corpus bursae large, elliptical; with two signa: one elliptical, with dense denticles, the other plate-shaped, bearing 3–6 teeth (Fig. 19a, b).

#### Distribution.

China (Chongqing, Henan, Hubei, Jiangxi, Shaanxi, Yunnan, Zhejiang).

#### Etymology.

The specific epithet is derived from the Latin *condensatus*, referring to the dense granules in the ductus seminalis.

### ﻿ Opacoptera ecblasta

Taxon classificationAnimaliaLepidopteraLecithoceridae

Wu, 1996

8C62980C-7DE7-5AE0-9E4A-AA5D50DB1174

Figs 5 , 13

Opacoptera ecblasta Wu, 1996: 12. TL: China (Sichuan). TD: IZCAS.

#### Material examined.

**China**: 1♂, Hubei, Xianfeng, Mahexiang, 400 m, 24.vii.1999, HH Li et al. leg., slide No. LSR11174; 3♂, Chongqing, Mt. Jinfo, 1100 m, 6–7.viii.2012, XF Yang & TT Liu leg.; 2♂, Chongqing, Mt. simian, 1280 m, 11–12.viii.2012, XF Yang & TT Liu leg.; 9♂, Guizhou, Mayanghe, 430 m, 5–10.vi.2007, XC Du leg., slide Nos. LSR11239, LSR11240, LSR14013; 1♂, 28.ix.2007, 1♂, 1.x.2007, Guizhou, Mayanghe, 700 m, H Zhen leg.; 3♂, Guizhou, Xishui County, 500 m, 24, 26.ix.2000, HL Yu leg.; 2♂, Guizhou, Xishui County, 500 m, 31.v.2000, YL Du leg., slide No. ZMR09041; 1♂, Guizhou, Chishui, Suoluo, 240 m, 23.ix.2000, HL Yu leg.; 1♂, Guizhou, Chishui, Suoluo, 390 m, 27.v.2000, YL Du leg.; 3♂, Yunnan, Malipo County, Xiajinchang, 1470 m, 26, 29.vii.2016, KJ Teng, GE Lee & T Wang leg., slide No. YS19423; 11♂, Yunnan, Daozhen County, Xiannvdong, 600 m, 17–18.viii.2004, YL Xiao leg., slide No. LSR13339.

#### Diagnosis.

This species is similar to *O.kerastiodes* Park, 2021 both in appearance and male genitalia. It can be distinguished by the third palpomere of the labial palpus with hair-pencils, the heart-shaped uncus and the needle-like posterior lobes of the juxta (Fig. 13); in *O.kerastiodes*, the third palpomere of the labial palpus is smooth, the uncus is subrectangular, and the posterior lobes of the juxta are horn-shaped.

**Figures 11–14.**
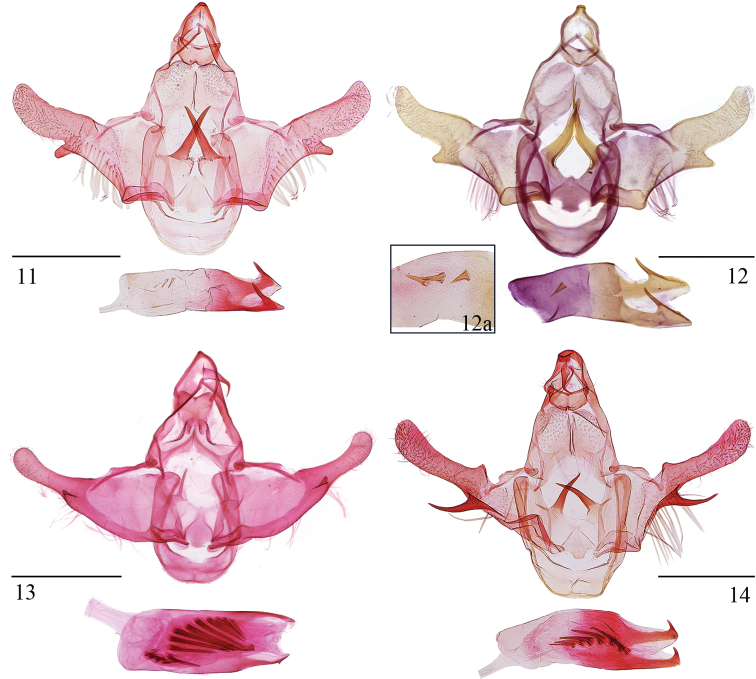
Male genitalia of *Opacoptera* spp. **11***O.callirrhabda* (Meyrick, 1936), slide No. YS19428 **12***O.condensata* sp. nov., holotype, slide No. LSR12090 **12a** cornuti of *O.condensata* sp. nov., paratype, slide No. YS17042 **13***O.ecblasta* Wu, 1996, slide No. LSR14013 **14***O.hybocentra* sp. nov., holotype, slide No. YS19424. Scale bars: 0.5 mm.

#### Description.

Wingspan 11.0–13.5 mm (Fig. 5).

#### Distribution.

China (Chongqing, Guizhou, Hubei, Sichuan, Yunnan).

### ﻿ Opacoptera flavicana

Taxon classificationAnimaliaLepidopteraLecithoceridae

Wu & Liu, 1992

F4A4661A-CD66-5D6B-AF6F-ABEF49C203C8

Figs 6 , 20

Opacoptera flavicana Wu & Liu, 1992 in Peng and Liu 1992: 618. TL: China (Hunan). TD: IZCAS.

#### Material examined.

**China**: 1♀, Guizhou, Mt. Fanjing, 1700 m, 1.vi.2002, XP Wang leg., slide No. LSR11247.

#### Diagnosis.

This species can be distinguished from its congeners by the pale yellowish brown forewing, the juxta without posterior lobes in the male genitalia (Wu and Liu 1992: 681, fig. 2148), and the corpus bursae with a single signum in the female genitalia (Fig. 20).

#### Description.

Wingspan 16.0 mm (Fig. 6).

#### Distribution.

China (Guizhou, Hunan).

#### Remarks.

Wu and Liu (1992) described the species from Hunan, China on a male and four females, and placed it in the genus *Opacoptera* based mainly on the wing venation. But the species seems distinctive in the genus *Opacoptera* both in appearance and genitalia as stated in the diagnosis. It probably does not belong to the present genus and the taxonomic status needs further study.

### ﻿ Opacoptera hybocentra

Taxon classificationAnimaliaLepidopteraLecithoceridae

Yu & Wang sp. nov.

00D29BBC-E8CC-59EF-92B4-9AA9E1BDFEA6

https://zoobank.org/B6432DFD-7F96-4055-BFC4-505F86CAD05C

Figs 7 , 14 , 21

#### Type material.

***Holotype*: China**: ♂, Yunnan, Baoshan, Nankang (24°49'N, 98°47'E), 2009 m, 20.vii.2015, KJ Teng & X Bai leg., slide No. YS19424. ***Paratypes*: China, Yunnan**: 39♂27♀, same data as holotype except dated 16–20.vii.2015, slide Nos. YS19431♂, YS19432♀; 1♂3♀, 10, 17.viii.2014, KJ Teng et al. leg., other same data as holotype; 2♂1♀, Longling County, Mt. Xiaohei, 1974 m, 18–19.vii.2013, SR Liu et al. leg., slide Nos. LSR13199♂, LSR13208♂, LSR13214♀; 1♂, Longling County, Mt. Xiaohei, 1974 m, 30.vii.2015, KJ Teng & X Bai leg.

#### Diagnosis.

The male genitalia of the new species is similar to that of *O.introflexa* sp. nov. It can be distinguished by the forewing having CuA_1_ and CuA_2_ separate and the horn-shaped ventrobasal process of the cucullus; in *O.introflexa* sp. nov., the forewing has veins CuA_1_ and CuA_2_ stalked and the ventrobasal process of the cucullus is broadly rounded, thumb shaped.

#### Description.

Wingspan 12.0–13.5 mm (Fig. 7). Head dark brown. Antenna with scape yellowish brown, flagellum pale brownish yellow. Labial palpus pale brownish yellow, third palpomere as long as second palpomere. Thorax and tegula dark brown. Forewing dark brown, with two black patches: one at basal 1/4, elliptical, the other at middle, shape ill-defined; fringe yellowish brown; CuA_1_ and CuA_2_ separate. Hindwing and fringe pale brownish yellow except yellowish brown around apical area.

***Male genitalia*** (Fig. 14). Uncus wide, shallowly concave on posterior margin, obtuse on anterior margin; caudal lobe papillary. Gnathos with basal plate obtuse on posterior margin; median process wide at base, narrowed to distal 1/3 where it curves, distal 1/3 spine-shaped. Valva wide basally, narrowed distinctly to cucullus; cucullus about half length of valva, parallel-sided in basal half, widened slightly to obtuse apex, costal margin shallowly concave except gently produced at base; ventrobasal process horn-shaped, curved, longer than basal width of cucullus; costal bar narrow, slightly expanded dorsad medially; sacculus wide, about 1/4 length of ventral margin of valva. Saccus obtuse on anterior margin. Juxta subquadrate, broadly concave on posterior margin, obtusely produced at middle on anterior margin; posterior lobe large, horn-shaped, nearly as long as juxta; posterolateral lobe short, spiniform, extending posterolaterally. Aedeagus slightly shorter than valva, slightly widened medially, with two dorsal denticles; cornuti consisting of six large, needle-like spines running from basal 1/3 to 2/3.

***Female genitalia*** (Fig. 21). Eighth abdominal sternite obtusely rounded on posterior margin. Apophyses posteriores about 1.5 times length of apophyses anteriores. Antrum funnel-shaped, wrinkled, membranous except sclerotized along posterior margin forming a band. Ductus bursae narrowed posteriorly, widened toward corpus bursae; ductus seminalis broad, roundly sac-like and bearing dense spinules basally. Corpus bursae ovate; signa consisting of six small teeth in a longitudinal row and a rounded, densely denticulate plate.

#### Distribution.

China (Yunnan).

#### Etymology.

The specific name is derived from the Latin *hybocentrus*, referring to the curved ventrobasal process of the cucullus.

### ﻿ Opacoptera introflexa

Taxon classificationAnimaliaLepidopteraLecithoceridae

Yu & Wang sp. nov.

03FAC9FD-DB81-51AD-8D39-EFC8483B45BD

https://zoobank.org/2B567F45-C597-4898-BEF4-F4A8682A337E

Figs 8 , 15 , 22

#### Type material.

***Holotype*: China**: ♂, Baoshan, Baihualing, Hanlongzhai (25°18'N, 98°49'E), 1577 m, 2.viii.2015, KL Liu & JX Zhao leg., slide No. YS19429. ***Paratypes*: China**: 1♂11♀, same data as holotype, slide No. YS19430♀; 9♂1♀, Yunnan, Gongshan County, Naqiutong Village, 1767 m, 16–18.vi.2017, KJ Teng et al. leg., slide No. YS19426♂.

#### Diagnosis.

The new species is unique among other species in the genus by having a triangular process at the base of the posterior lobe of the juxta at the inner margin. It is similar to *O.hybocentra* sp. nov., and the differences between them are stated in the diagnosis of the latter species.

#### Description.

Wingspan 13.0–13.5 mm (Fig. 8). Head dark brown. Antenna with scape brownish yellow; flagellum orange yellow. Labial palpus orange yellow except dark brown ventrally on third palpomere; third palpomere as long as second palpomere. Thorax and tegula dark brown. Forewing dark brown, with two, narrow black patches, one at the basal 1/4 and one in the middle, distal 1/3 with diffused black scales; fringe dark brown; CuA_1_ and CuA_2_ short-stalked. Hindwing and fringe brown.

**Figures 15–17.**
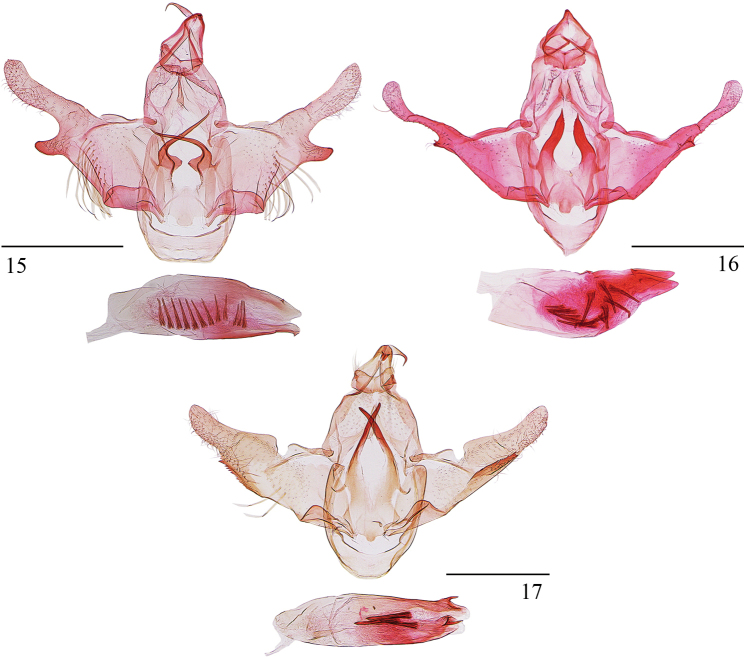
Male genitalia of *Opacoptera* spp. **15***O.introflexa* sp. nov., holotype, slide No. YS19429 **16***O.kerastiodes* Park, 2021, slide No. YS19048 **17***O.longissima* sp. nov., holotype, slide No. YS19422. Scale bars: 0.5 mm.

***Male genitalia*** (Fig. 15). Uncus subcrescent, broad V-shaped on posterior margin. Gnathos with median process slightly broad in basal 1/3, thereafter slendered to pointed apex, curved ventrad at distal 1/3 by a right angle. Valva wide in basal 1/4, narrowed distinctly to cucullus; cucullus about half length of valva, almost tubular, apex obtuse, costal margin shallowly concave, ventrobasal process thumbed; costal bar narrow, expanded dorsad medially; sacculus wide in basal half, slender in distal half, reaching cucullus. Saccus obtuse on anterior margin. Juxta subquadrate, broadly concave on posterior margin, densely denticulate along lateral sides of concavity; anterior margin obtusely produced at middle; posterior lobe large horn-shaped, longer than juxta, curved inward, triangularly produced at base on inner margin; posterolateral lobe small, spiniform. Aedeagus shorter than valva, slightly widened medially, with two tiny dorsal denticles; cornuti consisting of more than ten large, needle-like spines running from basal 1/4 to 3/4.

***Female genitalia*** (Fig. 22). Eighth abdominal sternite obtuse on posterior margin, with a sclerotized sac at anterolateral corner. Apophyses posteriores about twice length of apophyses anteriores. Antrum membranous except sclerotized along posterior margin forming a band, with two symmetrically sclerotized, leaf-like sclerites medially. Ductus bursae wrinkled, narrowed posteriorly, widened toward corpus bursae, with numerous conic spinules in anterior 3/5; ductus seminalis broad, arising from ductus bursae anteriorly, with sparse thorns on inner wall. Corpus bursae elliptical; signa consisting of several teeth of varied size in a longitudinal row placed posteriorly and a densely denticulate plate placed at middle.

**Figures 18–22.**
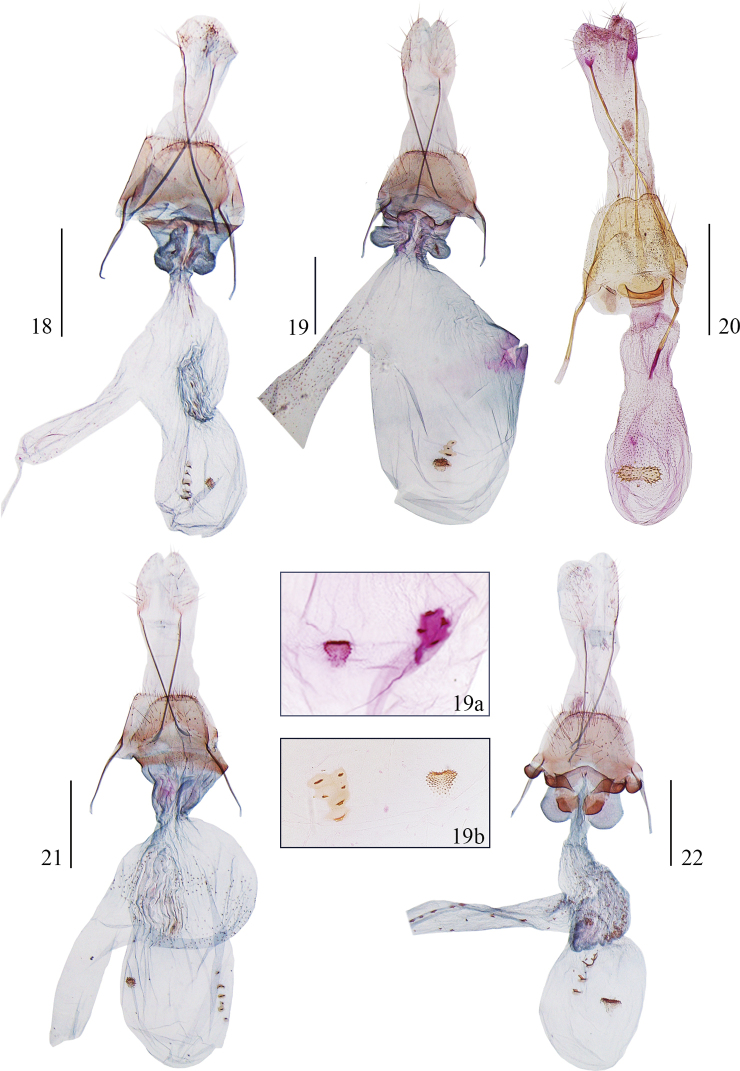
Female genitalia of *Opacoptera* spp. **18***O.callirrhabda* (Meyrick, 1936), slide No. YS19435 **19***O.condensata* sp. nov., paratype, slide No. YS19434 **19a** signa of *O.condensata* sp. nov., slide No. LSR11297 **19b** signa of *O.condensata* sp. nov., slide No. YS17057 **20***O.flavicana* Wu & Liu, 1992, slide No. LSR11247 **21***O.hybocentra* sp. nov., paratype, slide No. YS19432 **22***O.introflexa* sp. nov., paratype, slide No. YS19430. Scale bars: 0.5 mm.

#### Distribution.

China (Yunnan).

#### Etymology.

The specific epithet is derived from the Latin *introflexus*, referring to the medially curving posterior lobes of the juxta.

### ﻿ Opacoptera kerastiodes

Taxon classificationAnimaliaLepidopteraLecithoceridae

Park, 2021

DFC770F4-EFE0-5234-8B93-5767060D0635

Figs 9 , 16

Opacoptera kerastiodes Park, 2021 in Park and Kim 2021: 175. TL: Thailand (Chiang Mai). TD: ZMUC.

#### Material examined.

**China**: 1♂, 31.vii.2019, 2♂, 2.viii.2019, Yunnan, Menghai County, Damanlu Village, 1128 m, KJ Teng et al. leg., slide No. YS19048.

#### Description.

Wingspan 12.5–13.0 mm (Fig. 9).

#### Diagnosis.

This species is distinct among other species by having a clavate valva (Fig. 16). It is similar to *O.ecblasta* Wu, 1996, and the differences between them are stated in the diagnosis of the latter species.

#### Distribution.

China (Yunnan, new record), Thailand (Park and Kim 2021).

#### Remarks.

This species was originally described from Thailand based on a single male. It is recorded from China for the first time in this paper.

### ﻿ Opacoptera longissima

Taxon classificationAnimaliaLepidopteraLecithoceridae

Yu & Wang sp. nov.

41D1C175-6B8E-5A76-98DA-60F9326EE69C

https://zoobank.org/D99A862A-F378-4A30-A9EE-FA2A109FDAC8

Figs 10 , 17

#### Type material.

***Holotype*: China**: ♂, Yunnan, Tengchong, Cuanlong Village (25°19'N, 98°42'E), 1329 m, 10.viii.2015, KL Liu & JX Zhao leg., slide No. YS19422. ***Paratypes*: China**: 2♂, same data as holotype.

#### Diagnosis.

The new species can be distinguished from its congeners by the ventrobasally serrate cucullus and the apices of the posterior lobes of the juxta extending beyond the middle of the tegumen.

#### Description.

Wingspan 13.0–14.0 mm (Fig. 10). Head dark brown. Antenna orange yellow, paler distally. Labial palpus orange yellow except third palpomere dark brown ventrally; third palpomere as long as second palpomere. Thorax and tegula dark brown. Forewing dark brown, with diffused black scales distally; with two ill-defined black patches at basal 1/4 and middle respectively; fringe dark brown; CuA_1_ and CuA_2_ short-stalked. Hindwing and fringe brown; fringe with an orange white basal line.

***Male genitalia*** (Fig. 17). Uncus subrectangular, semicircularly concave on posterior margin; caudal lobe seimiovate. Gnathos with median process wide at base, narrowed to middle, slendered from middle to pointed apex, curved ventrad at distal 1/4 by a right angle. Valva wide at base, narrowed slightly to cucullus; cucullus about half length of valva, narrowed to obtusely rounded apex, costal margin expanded dorsad basally, ventral margin serrate ventrobasally and gently concave at middle; costal bar narrow, slightly arched, triangularly produced at middle on dorsal margin; sacculus narrow basally, widened distally, about 1/4 length of ventral margin of valva. Saccus rounded on anterior margin. Juxta deeply concave in V-shape on posterior margin, with a papillary process at middle on anterior margin; posterior lobe large horn-shaped, extending beyond middle of tegumen apically; posterolateral lobe small, narrowly banded, extending posterolaterally. Aedeagus nearly as long as valva, slightly widened medially, with two dorsal denticles; cornuti consisting of three large needle-like spines.

**Female** unknown.

#### Distribution.

China (Yunnan).

#### Etymology.

The specific name is derived from the Latin *longissimus*, referring to the long posterior lobe of the juxta.

## Supplementary Material

XML Treatment for Opacoptera

XML Treatment for Opacoptera callirrhabda

XML Treatment for Opacoptera condensata

XML Treatment for Opacoptera ecblasta

XML Treatment for Opacoptera flavicana

XML Treatment for Opacoptera hybocentra

XML Treatment for Opacoptera introflexa

XML Treatment for Opacoptera kerastiodes

XML Treatment for Opacoptera longissima
